# Notes and Notices

**Published:** 1888-12

**Authors:** 


### NOTES AND NOTICES.

REMOVALS.—Dr. Overton F. Macdonald, from Toronto to Wissahickon, Philadelphia; Dr. Chas. L. Dyer, from San José, California, to Baltimore.

HAHNEMANNIAN MONTHLY: FOR SALE.—Thirty-six volumes of this well-known periodical, unbound, for sale. They form part of the medical library of the venerable Dr. Adolphus Fellger, lately deceased. Apply to Mrs. Dr. Ad. Fellger, 154 N. Eleventh St., Philadelphia.

DR. LIPPE'S LIBRARY.—Catalogues of the medical library of the late Dr. Ad. Lippe are ready, and will be mailed upon application to W. C. Hall, Esq., executor, 251 S. Fourth St., Philadelphia.

DR. FAULKNER'S VISITING LIST for the new year has just been issued. It contains spaces for recording the prescription as well as the visit paid. In the forepart is a neat little repertory for ready reference in acute cases. Like so many other Visiting Lists, it is perpetual.

OTIS CLAPP & SON'S VISITING LIST for Physicians, comes along for the new year. It is not only a record of visits, but it has space for the noting of the remedy given. It is perpetual.

A JOURNAL OF OPHTHALMOLOGY, OTOLOGY, ETC.—In January next there will be published by A. L. Chatterton & Co., New York, *The Journal of Ophthalmology, Otology, and Laryngology.* It will be edited by George S. Norton, M. D., assisted by Chas. Deady, M. D. The editors have determined to make the journal of the highest practical value to all interested in the eye, ear, or throat. To accomplish this the mass of material to be found at the N. Y. Ophthalmological Hospital will be utilized, in addition to which there will be articles by prominent authorities throughout the country. The publication will appear quarterly, and consist of about four hundred pages at three dollars per year.

